# Prevalence of molar incisor hypomineralization among school children in Rome, Italy

**DOI:** 10.1038/s41598-022-10050-0

**Published:** 2022-05-05

**Authors:** F. Nisii, M. Mazur, C. De Nuccio, C. Martucci, M. Spuntarelli, S. Labozzetta, A. Fratini, S. Sozzi, A. Maruotti, I. Vozza, V. Luzzi, M. Bossu, L. Ottolenghi, A. Polimeni

**Affiliations:** 1 Paediatric Dentistry Unit, ASL Roma 1, Nuovo Regina Margherita Hospital, Rome, Italy; 2 Department of Oral and Maxillofacial Sciences, Sapienza University of Rome, Via Caserta 6, 00161 Rome, Italy; 3 Department of Mathematics, University of Bergen, Bergen, Norway

**Keywords:** Dentistry, Diagnosis, Paediatrics, Public health

## Abstract

Molar incisor hypomineralization (MIH) is a highly prevalent condition associated with increased caries experience, dental pain and treatment need. Aim of this study was to determine the prevalence and severity of MIH in a group of 7–8 years old primary school children living in Rome, Italy; and to assess the association with caries experience and possible perinatal risk factors. A survey has been conducted in the city of Rome, between April 2019 and March 2020 with a total of 49 primary schools and 176 2nd grade primary school classes and a total of 3611 children being involved. Of these, a subset of 346 children of 21 primary schools was selected for the epidemiological investigation. The prevalence of MIH was of 18.2%, with girls showing twice the probability of being subject to a mild-severe condition. Molar location was present in 71.4%, while location on both molar plus incisor was present in 28.6% of cases. The mean DMFT was 0.44 ± 0.78, “D” was 0.17 ± 0.58; the mean dmft was 1.7 ± 2.56, “d” was 1.32 ± 2.21. Female gender, caries experience, insufficient oral hygiene were risk factors. The incidence of MIH is increasing in the pediatric population. Knowledge about diagnosis and treatment options should be disseminated among dental professionals.

## Introduction

Molar incisor hypomineralization (MIH) is defined as “enamel qualitative developmental defect with systemic origin of one or more permanent first molars with or without incisor involvement”^1,2^.

Hypomineralization of molars and incisors is associated with an increased need for dental care, especially in severe cases^3–6^, with post-eruptive breakdown, increased porosity and development of carious lesions leading to pulpitis and hypersensitivity or pain^1,7–9^. The resulting pulpitis hinders local anesthesia^10^ and as a result, children with MIH show much greater anxiety and dental fear^4^. In addition, an aesthetic problem occurs when MIH affect also the upper arch teeth^11,12^ Generally, MIH negatively affects general health, quality of life and socio-psychological condition of children^6,7^.

Recently a systematic review determined the global burden of MIH as highly prevalent across the globe, with a mean estimated prevalence of 13.1%, affecting 878 million people. The new cases each year are 17.5 million and of these 27.4% will need therapy due to pain, hypersensitivity or post-eruptive breakdown^13^.

No recent data on MIH in Italian pediatric population is available in literature. The latest data was published in 2005 on a survey on 277 children with a reported MIH prevalence of 13.7%, with 5.8% on the first molars. All the reported MIH were mild form, except for one child with severe MIH^14^.

Aim of this study was to determine the prevalence of MIH in a group of 7–8 years old primary school children living in Rome, Italy, with severity classification according to MIH severity scoring system (MIH-SSS); secondary aim was to analyze the association with caries experience (dmft/DMFT) and to assess possible associated risk factors.

## Results

### Study population

Between April 2019 and March 2020, a total of 3611 children from 49 primary schools and 176 2nd grade classes was screened. Of these, a subset of 346 children of 21 primary schools was selected for an epidemiological investigation on the MIH prevalence and associated risk factors. The age of the enrolled children was 8 (SD ± 0.2) years old, of which 175 were female (50.6%) and 171 male (49.4%).

### MIH prevalence and location

The overall sample was of 346 children: a total of 63 cases (18.2%) with MIH was screened.

Molar location was present in 45 cases (71.4%), while location on both molar plus incisor was present in 18 cases (28.6%). Overall, mild form of MIH was in 11 cases (17.5%), while a severe form was present in 52 cases (82.5%). When molars were the unique location, 36 cases (80%) presented with a severe MIH form. When the location was both on molars and incisors, a severe form was shown in 16 cases (88.9%). None of the molars with MIH was classified as atypical restoration with/without marginal defect, extracted or not erupted (MIH-SSS code 7–10). A gender-based approach was used to analyze data on MIH in the whole sample and in the affected groups. Tables 1 and 2.

**Table1:** Gender-based analysis of MIH in the whole sample and in the affected groups.

Group	n	MIH	MIH molars	MIH molars and incisors	Mild	Severe
**All the group**						
Both	346	63 (18.2%)	45 (13.0%)	18 (5.2%)	11 (3.2%)	52 (15.0%)
Male	171	23 (13.5%)	19 (11.1%)	4 (2.3%)	4 (2.3%)	19 (11.1%)
Female	175	40 (22.9%)	26 (14.9%)	14 (8.0%)	7 (4.0%)	33 (18.9%)
**MIH present**						
Both	63	63 (100%)	45 (71.4%)	18 (28.6%)	11 (17.5%)	52 (82.5%)
Male	23	23 (100%)	19 (82.6%)	4 (17.4%)	4 (17.4%)	19 (82.6%)
Female	40	40 (100%)	26 (65.0%)	14 (35.0%)	7 (17.5%)	33 (82.5%)

**Table 2:** Gender-based analysis in cases of MIH with exclusive molar location and in those with molar + incisor location.

Group	n	Mild	Severe
**MIH molars only**			
Both	45	9 (20.0%)	36 (80.0%)
Male	19	3 (15.8%)	16 (84.2%)
Female	26	6 (23.1%)	20 (76.9%)
**MIH both**			
Both	18	2 (11.1%)	16 (88.9%)
Male	4	1 (25.0%)	3 (75.0%)
Female	14	1 (7.1%)	13 (92.9%)

### Caries experience

The mean DMFT was 0.44 ± 0.78, “D” was 0.17 ± 0.58; the mean dmft was 1.7 ± 2.56, “d” was 1.32 ± 2.21. Table 3 shows the distribution of DMFT/dmft among different groups (female, male, with/without MIH, with mild/severe MIH, etc.).

**Table 3:** Caries experience (DMFT/dmft) in the whole sample and in the different subgroups (female, male, with/without MIH).

	Min	Max	Mean	SD
**All**				
DMFT	0	4	0.263	0.789
dmft	0	12	1.682	2.566
**w/o MIH**				
DMFT	0	4	0.212	0.761
dmft	0	12	1.548	2.483
**MIH**				
DMFT	0	3	0.492	0.878
dmft	0	12	2.286	2.854
**Mild**				
DMFT	0	3	0.273	0.905
dmft	0	4	0.636	1.286
**Severe**				
DMFT	0	3	0.538	0.874
dmft	0	12	2.635	2.977
**Male**				
DMFT	0	4	0.251	0.760
dmft	0	12	1.719	2.673
**Female**				
DMFT	0	4	0.274	0.819
dmft	0	8	1.646	2.463
**Male, w/o MIH**				
DMFT	0	4	0.223	0.745
dmft	0	12	1.595	2.557
**Female, w/o MIH**				
DMFT	0	4	0.2	0.780
dmft	0	8	1.496	2.406
**Male, MIH**				
DMFT	0	3	0.435	0.843
dmft	0	12	2.521	3.273
**Female, MIH**				
DMFT	0	3	0.525	0.905
dmft	0	8	2.15	2.617

### Orthodontic treatment need

Orthodontic treatment need was evaluated with the IOTN Index. In the enrolled sample, 58% presented with a IOTN score > = 3, (score 3 = 15.7%, score 4 = 42.3%). Class II malocclusion was present in the 22.3%, class III in the 9.8% and 102 children (29.5%) presented a maxillary contraction with posterior bilateral cross bite in 49 cases.

### Perinatal factors

The geographic area of origin was in 89.9% European Community, 3.2% Philippines, 2.9% North Africa, 1.4% South America. China, States outside the European Community, India and South Africa were the 0.8%, 0.8%, 0.5% and 0.3%, respectively. Italy was in the vast majority of the sample the place of birth (95.6%), then India, Communitarian Europe, Extra communitarian Europe, South America and North Africa in 1.2%, 1.2%, 0.8%, 0.6% and 0.6% respectively.

Pre-term birth was reported by parents and caregivers in 20 children (5.8%). Breastfeeding was reported in 247 children (71.4%). Thirty-two (9.2%) presented allergies, while cardiac, respiratory and renal pathologies were reported in 2%, 0.8% and 0% of the sample, respectively. Diabetes type 1 was present in 0.6% of the whole sample.

### Lifestyle factors

Mean BMI was 16.3 ± 2.3, and it was collected on the 68.8% of the sample population, as it was a voluntary information the caregivers were invited to add to the informed consent.

The diet was varied for 91.3% of the enrolled population, 5.2% reported a free diet rich in sugar, and 2.3% has dietary restrictions for lactose intolerance. The reported oral habits over the 2 years of age were: thumb sucking (4.6%), pacifier use (18.8%), tongue sucking (2%), bottle use (20%), lip chewing (3.2%) and atypical swallowing (2%). Breathing was nasal in most cases (77.7%), oral in 12.7% and snoring at night in 8.7% of the sample. Forty-one children (11.8%) had systemic fluoride.

### Statistical analysis

The R statistical program, ver. 4.1.2 (The R Foundation for Statistical Computing, Wirtschaftsuniversität Wien, Vienna, Austria) was used for the statistical analyses. The results were considered statistically significant at *p* < 0.05.

### Logistic model

Factors influencing probability of MIH were selected from perinatal factors for etiology, gender, geographic area of origin. Geographic area of origin was restricted to Europe/non-Europe due to small number of patients from other geographical areas. Logistic regression was used, the optimal model was selected by Akaike information criterion value (AIC). The patient’s gender was the only variable in optimal model, it showed significant influence on the probability of MIH (Table 4). Log of odds ratio was 0.645 lower for male than for female patients. Factors influencing probability of severe form of MIH were also examined. No factors with significant influence were detected.

**Table 4:** Logistic model results for probability of MIH.

Variable	Estimate	Std. error	z-value	*p*-value
(Intercept)	− 1.216	0.180	− 6.757	< 0.001
Gender.male	− 0.645	0.288	− 2.245	0.025
Model characteristics	AIC: 327.18			
	McFadden’s R^2^ = 0.016			

Significance of the difference in DMFT, visible plaque and IOTN > 3 between groups without/with MIH is presented in the Table 5. DMFT and visible plaque were significantly larger in the group with MIH than w/o it (all the patients and female patients only). IOTN > 3 cases were significantly larger in the group with MIH than w/o it for male patients.

**Table 5:** Significance of the difference in DMFT, visible plaque and IOTN > 3 between groups without/with MIH.

	w/o MIH		MIH		*p*.value
	Mean	SD	Mean	SD	
**DMFT**					
Both	0.212	0.761	0.492	0.878	**0.021**
Male	0.223	0.745	0.435	0.843	0.265
Female	0.200	0.780	0.525	0.906	**0.044**
**Visible plaque**					
Both	0.2403	0.428	0.444	0.501	**0.004**
Male	0.2297	0.422	0.348	0.487	0.281
Female	0.2519	0.436	0.500	0.506	**0.007**
**IOTN > 3**					
Both	2.582	1.373	2.794	1.297	0.250
Male	2.313	1.379	2.870	1.180	**0.048**
Female	2.880	1.309	2.750	1.373	0.598

Significant values are in bold.

## Discussion

The present study aimed to represent actual condition on MIH among Italian children aged 7–8 years old in the urban area of Rome city. The enrolled population of 346 children was a subset of a Regional Health System ASL RM1 survey being conducted in 2019–2020 on a total of 3611 primary scholars attending to 2nd year course.

In 2005 data on MIH in Italian children population reported MIH prevalence of 13.7% and most detected lesions being mild form^14^_._ No data on Italian children are found in literature on associated risk factors and concomitant caries experience and/or orthodontic treatment need.

The present survey reported a MIH prevalence of 18.2% with most cases (82.5%) assessed as severe form with post-eruptive enamel breakdown with and without exposure of dentine. The results of the current study underline that MIH clinical condition is affecting now a larger number of Italian children with a more severe clinical form than in the past, with an increasing trend.

The current study aiming to report on MIH location, documented that in 71.4% it was exclusively on first molars and in 28.6% of cases on both molars and incisors. In accordance with our study population age, a previous epidemiological report showed that 8 year of age was considered as the best time for any examination for the MIH, as at this age, all 4 permanent molars will be erupted, as will be most of the incisors, while signs of MIH will be present^15^.

The classification used in this survey to assess the severity of MIH lesions was performed according to the MIH-SSS severity score system developed very recently by Cabral et al. ^16^. This latter is a severity scoring system with scores ranging from 0 to 10 (Table 6). Over the last 20 year two main diagnostic criteria for MIH classification have been proposed. The first one was proposed by the European Association of Pediatric Dentistry (EAPD) in 2003^15^ and was the most frequently used in MIH surveys. The second one was developed by Ghanim et al.^17^ and, accordingly to the Authors expectations, it should re-place the EAPD system. However, the index developed by Cabral et al.^16^ (MIH-SSS) was selected for this survey, as this latter firstly showed the survival curves for MIH enamel breakdown over time. In fact, Cabral index was developed over a period of 3 year of follow up, with a reliable evidence of MIH progression based on enamel color changes of MIH lesion. Cabral et al.^16^ documented that yellow/brown MIH opacities progressed more than did white/creamy opacities, with enamel post-eruptive breakdown being expected within 1 year.

The results of the current report showed that the vast majority of MIH cases (82.5%) were classified as severe form, with post-eruptive breakdown with/without exposure of dentine. Surprisingly no severe form of MIH was assessed as MIH-SSS code 7 and 8 (atypical restauration with/without marginal defect). The present screening study were not intended to collect data on access to dental care and therefore no data is available on any home or professional remineralization protocols that children may have undergone. A very recent study by Craveja et al. on knowledge and management of MIH among dentists and orthodontists in France, showed that large disparities about knowledge and management of MIH exist between dental practitioners, with 48% of the enrolled dentists who misdiagnosed MIH^18^. In accordance with the conclusions of this study, hypothesis may be raised upon the lack of treatment among Italian children presenting a severe form of MIH being assessed as untreated in the current survey, due to insufficient knowledge among dental professionals. Alanzi et al. conducted in 2018 a cross-sectional study to assess dentists’ knowledge on MIH: the results reported low levels of confidence in MIH diagnosis and the necessity of continuing education courses to provide high-quality dental care for children with MIH^19^.

The etiology of MIH is still unclear, but genetic and environmental factors have been proposed. Prenatal and early life period and genetic and epigenetic factors are thought to contribute^20^. In the current study a logistic regression model was considered to investigate possible determinants of the probability of observing MIH condition, with multivariable regressions analysis. Data on perinatal possible etiological risk factors collected during the survey were analyzed by the logistic regression model. None of the collected risk factor showed a significant statistical correlation with MIH condition. The limitation of cross-sectional studies is that the temporal association between the outcome and the exposure cannot be determined because both are examined at the same time. This drawback may be circumvented by formulating questions that assess the subject’s past, such as questions regarding previous lifestyle, occupation, or other exposures. In this study, a mandatory questionnaire was sent to parents prior to the dental screening visit; the data collected in this section on the children's medical history was dependent on the parents 'and caregivers' ability to remember and answer questions. A recent systematic review on the prevalence of MIH reported in 70 studies, showed no difference between genders^21^. Interestingly, the present survey showed that girls have almost twice the probability of being subject to a mild-severe condition than boys. Moreover, we estimated a significant increase in that probability for those having DMFT > 0, dmft > 0 and unsatisfactory /insufficient oral hygiene, with the latter having quite a strong impact. These results were in line with our expectations and with the existing literature^22^.

Caries experience is an important risk factor in children with MIH. In fact, a recent systematic review on the association of MIH and dental caries showed that children with MIH were 2.1–4.6 times more likely to have caries in the permanent dentition than children without MIH, with a strong association between the two clinical conditions^23^. In accordance with this review, the caries experience among the tested children was high with a mean dmft of 1.7 ± 2.56, “d” was 1.32 ± 2.21 and a mean DMFT of 0.44 ± 0.78, “D” was 0.17 ± 0.58.

Moreover, the presence of MIH is associated with dental anxiety and fear, hypersensitivity of the affected teeth and lack of collaboration in the oral hygiene procedures carried out at home.

In conclusion, in this clinical scenario, programs of oral screening among the primary school children population are of paramount importance together with dental education among dental professionals regarding knowledge about MIH diagnosis and management. Moreover, further research is needed to depict a clear etiology in order to start primary preventive measures.

## Material and methods

### Population

A survey has been conducted on the schoolchildren population in the central area of the city of Rome defined, accordingly to the Italian National Healthcare System, as ASL RM1, between April 2019 and March 2020 with a total of 49 primary schools and 176 2nd grade primary school classes and a total of 3611 children being involved. Of these, a subset of 346 children of 21 primary schools was selected in order to conduct an epidemiological investigation on the MIH prevalence and associated risk factors. This survey was part of the Lazio screening program for caries and malocclusion in 0–14 year old population, being conducted in close collaboration with the Italian National Healthcare System, as ASL RM 1.

The inclusion criteria were to be 7–8 year old and being a scholar of the primary school, attending to 2nd grade class.

### Sample size calculation

Sample size was calculated based upon the following formula:

$$n = p\left( {1 - p} \right)\left( \frac{Z}{e} \right)^{2}$$

where Z is the value from the standard normal distribution reflecting the confidence level (taken 95%), E is the desired margin of error (0.05), and p is the proportion of occurrences in the population. In the former study^14^ p was 0.137. But n increases while p nears to 0.5, so *p* = 0.333 was taken for safety. The sample size is of n = 342.

### Dental team and calibration

The dental team was composed of two dental professionals: one dentist and one dental hygienist skilled in epidemiological surveys. A total of three teams performed the dental screening. At T_0_ a calibration 3 day course was completed for the MIH diagnosis in the study, based on MIH-SSS scoring system. In the first phase, intraoral photographic images of MIH lesions in correlation with the classification system were used to illustrate each score. The operators were then asked to rate and assign a score to a new series of photographic images without clinical description. Inter-examiner reproducibility was calculated using the kappa statistic. The importance of rater reliability lies in the fact that it represents the extent to which the data collected in the study are correct representation of the variables measured. We found high values of the statistics for all considered variables, with kappa ranging between 0.93 and 0.96.

### Dental visit

Informed consent was obtained by all the children’s parents/caregivers, with agreement to collect and analyze the dataset obtained during the survey (Local Ethical Committee “Comitato Etico Lazio 1” n. paN-539). All research was performed in accordance with the Declaration of Helsinki. The screening was conducted at the primary schools, during the morning hours. All the children were invited to clean their teeth before the visit. Screening conditions were standardized: a portable dental chair with a dental light and a single use survey kit (mirror and probe) were used. The visit was performed by the dentists (MM, CM, MS) while the data was registered by the dental hygienist (SS, AF, SL) and collected on the software used for the dental screening.

### Recording of defects

The Molar Incisor Hypomineralization Severity Scoring System (MIH-SSS) was used to record the data on MIH (Cabral et al.^16^). Table 6 shows the MIH-SSS codes and its clinical description and the correspondence with the MIH Survey Severity Classification used in this survey.

**Table 6:** MIH classification used in the survey based on MIH-SSS scale.

MIH-SSS code	Clinical description	MIH severity classification used in the survey
0	No changes	–
1	Change in enamel color: white/creamy	Mild form
2	Change in enamel color: yellow/brown	Mild form
3	PEB with white/creamy enamel color	Severe form
4	PEB with yellow/brown enamel color	Severe form
5	PEB with exposed hard dentine	Severe form
6	PEB with exposed soft dentine	Severe form
7	Atypical restoration without marginal defect	DMFT
8	Atypical restoration with marginal defect	DMFT
9	Extracted	DMFT
10	Not erupted	DMFT

Age and gender were also recorded. MIH localization was assessed on molars exclusively or on molars + incisors.

### Data collection

Dental situation was evaluated, and the following data was recorded: (1) caries or caries experience prevalence: DMFT/dmft > 0; (2) Overall oral hygiene condition (very good, sufficient, insufficient); with (a) plaque (yes/no); (b) calculous (yes/no); (c) recession (yes/no); (d) fluorosis (yes/no); (e) gingivitis (yes/no); (f) bleeding on probing index (BPI).

The orthodontic situation was evaluated and the following items collected: (1) dental malocclusion (I, II, II-2, III class of malocclusion); (2) maxillary contraction (yes/no); (3) crossbite (anterior, posterior, posterior-monoloteral, posterior-bilateral); (4) crowding (= < and =  > to 4 mm); (5) OVJ and OVB (normal = 1–3 mm; increased > 3 mm; reduced < 1 mm); (6) DMJ signs and symptoms and (7) Orthodontic Treatment Need Index (IOTN Index), with scores > 3 indicating a need of orthodontic treatment.

### Etiological factors

The mandatory questionnaire fulfilled by the parents/caregivers was aimed to collect additional anamnestic data concerning prenatal, perinatal and postnatal etiological factors.

Prenatal factors: geographical area of origin; perinatal factors: preterm birth, breastfeeding; postnatal factors: breastfeeding, renal pathologies, allergies, cardiac pathologies, diabetes 1, respiratory pathologies, systemic fluoride. Moreover, data on (1) breathing; (2) oral habits and (3) diet was collected.

Table 7 shows the etiological factors collected during the survey.

**Table 7:** Possible etiological factors collected during the survey from the questionnaire fulfilled by the parents/caregivers.

Etiological factor	Prenatal	Perinatal	Postnatal
Geographical area of origin:	European area		
	Extra-European area		
Preterm﻿ birth			
Breastfeeding		Yes/no	Yes/no
Renal pathologies			Yes/no
Allergies			Yes/no
Cardiac pathologies			Yes/no
Diabetes 1			Yes/no
Respiratory pathologies			Yes/no
Systemic fluoride			Yes/no

### Statistical analysis

The main outcome was the presence of MIH, that is a binary variable. Multivariable logistic regressions models were considered to investigate possible determinants of the probability of MIH presence, and of the probability of observing severe condition if MIH is present. The preferred model was selected in a stepwise approach according to the Akaike Information Criterion.

### Ethical approval

Ethical approval was obtained from the Local Ethical Committee “Comitato Etico Lazio 1” (n. paN-539).

### Consent to participate

Written consent was obtained by the caregivers and/or parents of the study participants.

## Data Availability

Data are available upon request.
